# Effects of feeding sodium metabisulfite-treated fruit and vegetable discards to Hanwoo heifers and cows

**DOI:** 10.5713/ab.21.0244

**Published:** 2021-08-21

**Authors:** Won Hee Lee, Farhad Ahmadi, Young Il Kim, Jong Moon Park, Wan Sup Kwak

**Affiliations:** 1 Food Bio-science Major, College of Medical Life Sciences, Konkuk University, Chungju 27478, Korea; 2 Daepoong Co. LTD, Anyang 13930, Korea

**Keywords:** Growth Performance, Nitrogen Efficiency, Preservative, Ruminant Feeding, Soluble Carbohydrate, Waste Recycling

## Abstract

**Objective:**

Two series of experiments were conducted to determine how the incremental levels of sodium metabisulfite (SMB)-treated fruit and vegetable discards (FVD) in diet of Hanwoo heifers and cows affect their performance and health.

**Methods:**

In Exp. 1, 36 Hanwoo heifers were stratified by age (13.3±0.83 mo) and initial body weight (305±19.7 kg), and divided randomly to one of three diets containing 0%, 10%, or 20% SMB-treated FVD (as-fed basis). The experiment lasted 110 d, including 20 d of adaptation. In Exp. 2, 24 multiparous Hanwoo cows were divided into three groups based on age (48.2±2.81 mo) and initial body condition score (2.64±0.33). Cows in each block were assigned randomly to one of three diets containing 0%, 11%, or 22% SMB-treated FVD (as-fed basis). The experiment lasted 80 d, including a 20-d adaptation period. In both experiments, SMB-treated FVD was used as a replacement for wet brewers grain in total mixed ration (TMR).

**Results:**

Growing heifers exhibited no differences in their daily feed intake (6.58±0.61 kg/d dry matter [DM]), average daily gain (0.60±0.07 kg/d), and body condition score when they consumed the incremental levels of SMB-treated FVD. Although most blood metabolites were unaffected by treatments, blood urea-N and β-hydroxybutyrate levels decreased linearly as the SMB-treated FVD level increased in TMR. Similar to Exp. 1, minor differences were found in daily feed intake (8.27±0.72 kg DM/d) and body condition score of Hanwoo cows. Most blood metabolites remained unaffected by treatments, but blood urea-N decreased as the SMB-treated FVD level in TMR increased.

**Conclusion:**

Our findings suggest that SMB-treated FVD could be safely incorporated into the diet of Hanwoo heifers and cows, potentially improving N-use efficiency in the body while not impairing performance or health.

## INTRODUCTION

The global demand for fruit and vegetable production is increasing as a result of rising public health awareness, encouraging consumption of foods with health-promoting properties [1]. Substantial progress in harvesting and processing automation has aided in significant advances in handling and sorting systems over the last few decades [2]. However, a high incidence of damage still occurs during the processing stages, which reduces the shelf-life of fruits and vegetables and results in a significant amount of fruit and vegetable discards (FVD) in packinghouses. Although the Food Recovery Hierarchy has proposed several routes for the re-utilization of food wastes such as FVD, their recycling through livestock feed is the most preferable option [3]. We recently included incremental levels of sodium metabisulfite (SMB)-preserved FVD (0% to 30%, as-fed basis) in a mixed diet of sheep and found no negative effects on diet palatability or overall health [4]. Romero-Huelva et al [5] also found that replacing 35% of cereal-based concentrate with tomato and cucumber waste fruits in the diet of lactating goats had no negative effect on productivity, resulting in an improvement in the net food production index.

Our extensive research revealed that FVD contains a high concentration of water-soluble carbohydrates in addition to a high moisture content (WSC; 44% to 63% of dry matter [DM]) [3,4,6,7]. As a result, FVD biomass is extremely susceptible to rapid decomposition by both exogenous and endogenous microorganisms [3,7]. Previously, we confirmed the effectiveness of SMB at the rate of 6 g/kg FVD biomass in preserving the nutritional quality and antioxidant constituents of FVD under aerobic storage for at least a wk in a series of small- and large-scale experiments [3,4,6]. The safe storage of FVD within 7 d is critical because this is the typical accumulation period of FVD in the packinghouse center before they are transported to a recycling facility [3]. The high moisture content of FVD is another issue that must be addressed before considering FVD biomass as a feed source. High-moisture biomass poses several storage, handling, and transportation challenges, in addition to seepage production, which is an environmental hazard. These concerns may be minimized if FVD is combined with dry feed ingredients in the form of total mixed ration (TMR) [8].

Wet brewers grain (WBG) is a typical high-moisture feed ingredient in TMR of dairy and beef cattle because it is a good source of energy for ruminants (estimated total digestible nutrients [TDN] = 78%; [9]). Based on the moisture and energy content similarities between FVD (an estimated average TDN of 80.2% and moisture content ranging from 85% to 88% [3,4,6,7]) and WBG, we hypothesized that FVD could be used as a replacement of WBG in the rations of Hanwoo heifers and cows. Therefore, the primary objective of Exp. 1 was to examine how the different levels of SMB-treated FVD in TMR (0%, 10%, and 20% as-fed basis) in replacement to WBG would affect the diet palatability, growth rate, and health of growing Hanwoo heifers. In Exp. 2, the level of SMB-treated FVD in TMR was slightly increased in order to determine the potential effects on feed intake and health of Hanwoo cows.

## MATERIALS AND METHODS

### Collection of fruit and vegetable discards and sodium metabisulfite treatment

Before FVD collection, their individual ingredient proportion in total discards was surveyed and those ingredients constituting over 90% of total discards were selected [7]. The discards were collected every 20 d from the E-mart Logistics Fresh Center (Icheon, Korea), ground with a meat mincer, and mixed with SMB at a rate of 6 g/kg biomass (wet basis). The discards were stored in an aerobic environment for 7 d to simulate the field situation in the packinghouse center, where these discards accumulate until they are transported outside the center, usually lasting about 1 wk [3]. After 7 d of aerobic exposure, the FVD biomass was transferred into sealed polybags and stored anaerobically until it was added to the TMR.

### Experimental design and animal management

Animal-related experiments were permitted by the Animal Ethics Committee of Konkuk University under the approval numbers KU19221 (Exp. 1) and KU19222 (Exp. 2). In Exp. 1, 36 Hanwoo heifers were blocked by age (13.3±0.83 mo) and initial body weight (305±19.7 kg), and within each block were divided at random into one of three treatments (6 heifers/pen; 2 pens/treatment). The treatments were a control diet with no SMB-treated FVD or a diet with 10% or 20% SMB-treated FVD (as-fed basis), corresponding to 0%, 25%, or 50% WBG replaced by SMB-treated FVD, respectively. The experiment lasted 110 d, including a 20-d period of transition and adaptation to the experimental diets. Diets were provided as a TMR once daily at 7:00 a.m. In a TMR mixer, the dietary ingredients were thoroughly mixed for about 10 min. The chemical composition of individual ingredients used in the formulation of TMR in Exp. 1 and 2 are listed in Tables 2 and 9, respectively. Feed intake was recorded daily following the collection of refusal amounts prior to morning feeding. To minimize day-to-day variation, body weights were recorded about 1 h before feeding on two consecutive days, the first (d 1 of the experiment, which corresponds to d after the adaptation period ended) and last d (90) of the experiment.

In Exp. 2, 24 multiparous Hanwoo cows (age = 48.2±2.81 mo) were blocked by age and initial body condition score (2.64±0.33), and within each block, randomly divided into one of three treatments (4 cows/pen; 2 pens/treatment). Treatments included a diet with no SMB-treated FVD (control), or a diet containing 11% or 22% SMB-treated FVD (as-fed basis), equaling to 0%, 33%, and 67% WBG being replaced by SMB-treated FVD, respectively. The experiment lasted 80 d, including 20 d of transition and adaptation to experimental diets. Diets were fed twice daily as a TMR (07:00 and 18:00 h). All animals had free access to fresh water. In both experiments, behavioral events were recorded using closed-circuit television cameras (ISAW Inc., Seongnam, Korea). Individual cameras were installed at 2.2 m height at the front and rear sides of each pen, allowing the entire pen to be recorded. Recordings were made from 0700 to 1800 for three consecutive d (from d 86 to 88 in Exp. 1 and 56 to 58 in Exp. 2). Observations included eight categories: feeding, rumination, lying, standing, chewing, drinking, urination, and defecation frequencies.

### Sampling and analytical methods

After preparation of a water extract of FVD or dietary ingredients, the resultant filtrate was subjected to centrifugation (20 min at 10,000 *g*) and analyzed for WSC [10]. NH_3_-N was quantified in FVD biomass using the method of Barker and Summerson [11]. Using standard spread-plating methods, total bacteria, lactic acid bacteria, yeast, and mold were counted [7]. Dry matter, ether extract, crude protein (CP), ash, and ash-corrected neutral detergent fiber (NDF) (inclusive of alpha amylase) and acid detergent fiber (ADF) contents were determined using the AOAC methods [12]. Lignin was quantified in FVD biomass after cellulose solubilization with sulphuric acid (72%).

On two consecutive d before the termination of each experiment, near 4 h after delivering the morning feeding, blood was sampled by jugular venipuncture into evacuated tubes without or with anticoagulant (BD Vacutainer, Franklin Lakes, NJ, USA). Serum was harvested through centrifugation (1,200 *g* for 20 min). The harvested blood in the vacutainer tubes was immediately sent to the laboratory (Green Cross Corporation, Yongin, Korea) for the analysis of selected blood items.

### Data analysis

The data were analyzed with a completely randomized block design using the Proc Mixed function in SAS (version 9.3; SAS Institute Inc., Cary, NC, USA). Before analysis, the normality of data was checked using the Proc UNIVARIATE procedure of SAS. The model for analysis was as follows:


$${\text{Y}}_{\text{ijk}}=\mu +{\text{T}}_{\text{i}}+{\text{A}}_{\text{ij}}+{\text{P}}_{\text{k}}+{\text{BW}}_{0}+{ɛ}_{\text{ijk}}$$

where Y, μ, T, A, P, BW_0_ and ɛ represent the observation, mean, treatment effect, random effect of animal, period effect (for the data repeated over time), initial body weight as covariate, and error. Those data repeated over time were analyzed using Proc Mixed of SAS, with AR (1) variance–covariance structure included in the model. The SAS’s CONTRAST statement was also used to identify linear and quadratic effects. The Tukey’s test was used to distinguish treatment difference between least-squares means. The significance threshold was set at less than 0.05 probability.

## RESULTS AND DISCUSSION

### FVD preparation and diet characteristics

The nutrient composition and microbial population of SMB-treated FVD after 7-d aerobic preservation in Exp. 1, are presented in Table 1. During the 80-d collection period, DM, CP, and WSC contents ranged from 12.8% to 15.8%, from 6.1% to 8.9% of DM, and from 37.9% to 50.3% of DM, respectively. These values are consistent with those obtained in our laboratory over a 3-yr period, indicating that FVD biomass is high in soluble sugars and moisture but low in fiber and protein contents [3,4,6,7]. Our previous observations confirmed that these FVD biomass characteristics accelerated putrefaction by active microbial populations. This was the primary reason we used SMB as a preservative to delay microbial degradation of the biomass. After 7 d of aerobic exposure, the population of yeast or mold was near or below the detection level, indicating the effectiveness of SMB in suppressing the growth of these organisms [3,7]. Counts of LAB were typically below the detection level, indicating their high vulnerability to SMB.

With increasing SMB-treated FVD level in TMR, CP and DM contents slightly decreased (Table 3), which is due to the lesser CP and higher moisture content of FVD than WBG. However, compared with control, non-fibrous carbohydrates (NFC) and WSC concentrations increased by 2.70- and 2.33-unit percentage in TMR with 20% SMB-treated FVD, owing to the substantially higher content of NFC (average of 72.7% of DM) and soluble sugars (average of 43.6% of DM) in FVD than in WBG (Table 1). The majority of soluble sugars is removed during the malting and mashing processes, thereby resulting in a very low concentration of WSC in WBG [13]. The energy content of diets differed only slightly (average TDN = 60.2%).

### Performance of Hanwoo heifers (Exp. 1)

Feed DM intake and growth performance of Hanwoo heifers are reported in Table 4. Heifers assigned to the three experimental groups consumed similar amount of feed DM daily (6.58±0.61 kg/d DM; p = 0.31) and grew at comparable rates over the course of the 90-d experiment (0.60±0.07 kg/d average daily gain; p = 0.74). The lack of a treatment effect on feed intake suggests that incorporating SMB-treated FVD into TMR had only a minor effect on diet palatability by heifer. In line with this observation, Song et al [14] designed a 3-mo experiment using growing Hanwoo steers (initial BW of 287 kg; age = 9.1 mo) and identified minor differences in their daily DM intake and growth rate when they were offered a TMR without or with 20% of SMB-treated FVD (as-fed basis). A sheep metabolism experiment also identified no negative effect on voluntary feed consumption when SMB-treated FVD was included in the diet up to 30% (as-fed basis) [4]. Generally, the use of SMB as a silage additive improves the palatability of the resultant silage by beef or dairy cattle. For example, Cordukes and Shearer [15] reported that the low- to medium-DM forages (mainly alfalfa, red clover, and timothy) ensiled with SMB as silage modifier (3.63 g/kg forage) improved the acceptability of the silage by heifers. Smith and Campbell [16] conducted a study with dairy cows in which they gave cows free access to SMB-treated (4.5 g/kg biomass) and untreated silages, and they observed an evident preference of dairy cows to SMB-treated silage.

The minor effect of treatments on body weight gains is possibly related to the negligible differences in CP and energy intake among experimental diets because daily DM intake was not different among heifers. Contrary to our expectation that the increased WSC level in TMR did not improve heifer growth rate, Lee et al [17] reported that suckling lambs grazing on high-WSC forages gained more liveweight, which was explained by the synchronized supply of energy and nitrogen to the ruminal microorganisms. Soluble carbohydrates are a readily available source of energy for ruminal microorganisms, which is associated with increased microbial protein synthesis, a shift in ruminal fermentation patterns favoring an increase in the glucogenic: lipogenic volatile fatty acid ratio, and thus promoting ammonia and CH_4_ production reduction [18].

### Blood metabolites of Hanwoo heifers (Exp. 1)

Although the majority of blood metabolites were unaffected by the treatments, blood urea-N (BUN), total ketones, β-hydroxybutyric acid (βHBA), and sodium levels decreased linearly with inclusion of SMB-treated FVD in TMR (Table 5). Normally, the mean values for the majority of blood chemistry and hematological parameters are within the ranges reported in Hanwoo heifers of the same age [19]. The availability of carbohydrates in the rumen directly controls the growth rate of microbes in the rumen. Higher microbial protein efficiency in the rumen may be manifested by a decrease in BUN level, collectively contributing to increased N-use efficiency in ruminants [20]. Lower BUN concentrations were expected in heifers consuming SMB-treated FVD because the progressive levels of SMB-treated FVD in TMR resulted in a 2.3% unit increase in TMR WSC content (20% SMB-treated FVD) as compared to control TMR. Supporting this observation, Osborne et al [21] confirmed that adding glucose to drinking water of dairy cows (at rates of 10 and 20 g/L of water) resulted in a significant decrease of BUN level, which was translated to the more efficient use of dietary protein. The authors proposed that WSC can be easily used to maximize ammonia-N utilization by ruminal microorganisms. Previous research found that feeding sugars to sheep and dairy cows reduced N loss through urine but increased N retention, indicating an improvement in N-use efficiency [18,22]. However, the ratio of WSC to CP is more important than WSC concentration and has a significant impact on ruminant N-use efficiency. Edwards et al [23] identified some inconsistencies in N-use efficiency as WSC content increased, which was explained by the minor differences in WSC:CP ratio, despite large differences in WSC concentrations. Several studies in dairy cows suggested that any increase in the WSC:CP ratio improved the efficiency of N utilization and decreased urinary N losses [23]. In the current study, increasing the dietary level of SMB-treated FVD resulted in an increase in WSC:CP ratio (from 0.39 in control TMR to 0.60 in TMR containing 20% SMB-treated FVD), which explains the obvious difference in BUN concentration between treatments. More research is needed to quantify the amounts of N excreted via fecal and urinary routes, which would provide more evidence about the potential role of the SMB-treated FVD in improving ruminant N-use efficiency.

Lower levels of βHBA and total ketones in the blood of heifers fed SMB-treated FVD may indicate that the heifers’ energy status has improved. Blood glucose and βHBA are two of the most reliable indicators of energy metabolism in ruminants. For example, a negative correlation has been established between βHBA level and energy balance in dairy cows [24]. Presently, the energy level of diets was not different (a mean TDN of 60.2% of DM), signifying that the heifers received the similar level of energy. This observation could also be explained by the increased availability of soluble sugars in SMB-treated FVD-fed groups, which likely provided heifers with a readily available source of energy and thus improved their energy status. In support, Grummer et al [25] reported that the oral administration of propylene glycol (0, 296, 592, or 887 mL) as a glucogenic supplement in feed-restricted heifers resulted in a linear increase in plasma glucose and a linear decrease in plasma βHBA concentration. In a short-term metabolism experiment with sheep, however, we found no effect of progressive levels of SMB-treated FVD (from 0% to 30% TMR; as-fed basis) on blood metabolites including BUN. The lack of difference identification is likely related to the small number of animals and short experimental periods (28-d periods in a Latin square design), which may have limited the power of statistical analysis to identify the potential differences [4].

Heifers consuming the increased level of SMB-treated FVD in their diet exhibited no effect on blood copper and ceruloplasmin levels, which was an interesting finding given that a higher S load in the diet is known to impair copper bioavailability. When sulfur compounds degrade in the rumen, sulfide is formed, which readily reacts with copper to form copper sulfide, reducing copper bioavailability. The reduction in ceruloplasmin level is thought to be caused by a decrease in plasma copper level [26]. However, such effects were not identified in the present experiment. Although there is no experimental evidence to support this explanation, the low availability of S originating from SMB may have had a minor impact on S metabolism in the rumen. Furthermore, the partial loss of SMB to SO_2_ during aerobic storage [3] and the conversion of free sulfite to bound sulfite during storage and TMR production may have all contributed to the reduced ruminal availability of S sources derived from SMB.

Small treatment effects were found in hepatic-originating enzymes, which implies that the addition of SMB-treated FVD in the diet had no negative effect on liver metabolism and health. However, because liver enzymes are highly variable in blood, they have a low diagnostic value for nutritional status and should be interpreted with caution. Overall, the negligible variations in hematological and liver enzymes among treatments might suggest that SMB-treated FVD can be safely included up to 20% in the diet of Hanwoo heifers without adversely affecting their growth and health status.

### Behavior of Hanwoo heifers (Exp. 1)

As presented in Table 6, minor differences were detected in the behaviors of Hanwoo heifers when they consumed the different levels of SMB-treated FVD in their diet. However, eating time in heifers receiving TMR with 20% SMB-treated FVD tended to be the longest (155 min/11 h; P = 0.09). This could be explained by the increased SMB load in this group, which may have had a minor negative effect on TMR palatability in heifers. Previous studies yielded inconclusive results regarding the preference response of animals to the SMB-incorporated diets. For example, consistent with our findings, Meiske et al [27] fed pregnant beef cows corn silage with or without SMB (4 g SMB/kg biomass) and observed their slower consumption rate in SMB-treated than untreated silages. They reported that it took nearly 8 h for the cows to eat their morning meal, whereas it took approximately 4 h for the cows to consume control silage in the morning feed. Contrarily, in a dairy cow study, Smith and Campbell [16] observed a distinct preference of immature or mature silages treated with SMB (4.5 g/kg biomass) when given a free-choice access. The forage type and maturity, SMB application rate, and animal breed and growth stage appear to belong to the factors that influence the relative palatability of SMB-containing diets.

### Hanwoo cow experiment (Exp. 2)

Although the ingredient composition of FVD differed from that of Exp. 1, minor differences in the chemical composition of total discards were observed between the two experiments, with DM, NDF, CP, NFC, and WSC concentrations averaging 14.7%, 16.4%, 7.20%, 70.4%, and 52.1% of DM, respectively (Table 7). Similar to Exp. 1, increasing the inclusion of SMB-treated FVD in TMR increased NFC and WSC contents while slightly decreasing DM and CP contents (Table 8).

### Performance and blood metabolites of Hanwoo cows (Exp. 2)

Minor differences existed in the body condition score of Hanwoo cows before and after the experiment (Table 10). Body condition score employs a scaling method to estimate the energy reserves in the form of muscle and fat in cattle, and thus is a reliable reflection of the plane of nutrition over time [28]. Therefore, the lack of detectable differences in body condition score suggests that diets only had a minor impact on the nutritional status of Hanwoo cows.

Similar to Exp. 1, treatments had also slight effects on the majority of blood chemistry and blood hematological parameters (Table 11). However, similar to Exp. 1 a linear reduction was identified in BUN level as inclusion of SMB-treated FVD increased in the diet. Contrary to the findings of Exp. 1, we identified the minimal treatment effects on βHBA and total ketones. For unknown reasons, blood chlorine levels decreased linearly in group of cows receiving 22% SMB-treated FVD. Our review of the literature found no studies to have investigated the effects of FVD preserved with SMB on cattle blood metabolites; therefore, we were unable to compare the results of this experiment to previous reports. Regardless of treatment effect, there were some differences in blood metabolites between the two experiments. For example, higher phosphorus and free fatty acids but lower glucose and alkaline aminotransferase were quantified in blood samples from heifers than cows. The differences in physiological status, age, and diet composition may help to explain such differences because these factors are known to affect blood chemistry and hematology in livestock animals [29].

Treatments had no effect on Hanwoo cow behavior (Table 12). Contrary to our observation in Exp. 1 that eating time tended to be fastest in heifers fed 10% SMB-treated FVD in their TMR, there was no treatment effect on eating behavior of Hanwoo cows. Interestingly, Hanwoo cows spent slightly more time on eating behavior relative to Hanwoo heifers in Exp. 1. Seasonal climatic conditions (temperature and humidity), age, and pregnancy stages are among the factors that influence animal feeding behavior, which may explain some of the differences in animal behavior observed in the two experiments.

## CONCLUSION

Supplementation of SMB-treated FVD in the diets of Hanwoo heifers (up to 20%) and Hanwoo cows (up to 22%) had only minor effects on their daily feed intake. Heifers fed the three different diets grew at the similar rates. In both studies, small variations in hematological and hepatic enzymes were found, indicating that SMB-treated FVD did not have a negative impact on overall health of animals. In both trials, urea nitrogen in blood decreased linearly as the level of SMB-treated discards increased in the diet, which likely suggests the increased efficiency of nitrogen utilization, presumably due to the higher soluble-sugar content of the diets. Overall, our results support the safe use of SMB-preserved FVD by Hanwoo heifers and cows, which is important from both an economic and environmental standpoint.

## Figures and Tables

**Table 1:** Ingredient and nutrient composition of sodium metabisulfite-treated fruit and vegetable discards used in dietary treatments (Exp. 1; values in parentheses indicate standard deviation)

Items	Collection days^1)^				

	d 0	d 20	d 40	d 60	d 80
Individual ingredients (%)					
Grape	18.5	12.0	6.50	12.7	0
Apple	14.3	14.7	8.80	6.60	0
Plum	0	0	12.7	0	9.10
Avocado	0	0	4.70	6.24	0
Orange	13.4	15.1	0	0	11.2
Garlic	0	3.60	4.10	0	0
Sweet potato	12.0	9.20	2.80	8.10	0
Potato	4.49	0	13.9	7.84	6.40
Cucumber	0	0	6.80	6.10	0
Tomato	14.5	12.0	15.3	9.11	8.10
Onion	11.5	16.3	0	0	14.2
Lemon	0	6.80	6.50	6.70	5.60
Green onion stalk	7.20	5.10	0	5.30	0
Melon	0	5.20	5.10	9.50	14.2
Paprika	4.11	0	7.50	0	16.2
Pumpkin	0	0	5.30	6.19	6.50
Mushroom	0	0	0	3.31	4.20
Radish	0	0	0	5.01	4.30
Carrot	0	0	0	7.30	0
Nutrient composition					
DM (%)	15.8 (0.13)	14.4 (0.17)	12.8 (0.21)	13.5 (0.15)	13.7 (0.25)
NDF (% of DM)	13.7 (0.41)	14.4 (0.45)	12.9 (0.24)	12.1 (0.37)	16.7 (0.61)
ADF (% of DM)	9.45 (0.14)	9.68 (0.21)	9.44 (0.12)	7.90 (0.14)	12.5 (0.22)
Lignin (% of DM)	2.83 (0.12)	2.41 (0.13)	2.30 (0.09)	2.06 (0.11)	2.97 (0.14)
Ether extract (% of DM)	1.69 (0.10)	1.71 (0.08)	1.38 (0.12)	1.40 (0.07)	1.90 (0.11)
Crude protein (% of DM)	6.11 (0.51)	6.28 (0.33)	7.23 (0.28)	7.09 (0.42)	8.92 (0.46)
Ash (% of DM)	4.23 (0.26)	4.18 (0.18)	4.97 (0.15)	4.80 (0.19)	5.25 (0.21)
NFC (% of DM)	74.4 (0.63)	73.5 (0.77)	73.6 (0.53)	74.8 (0.73)	67.3 (0.67)
NH_3_-N (% of total N)	6.12 (0.34)	9.32 (0.49)	8.03 (0.61)	10.4 (0.31)	6.89 (0.39)
WSC (% of DM)	48.8 (2.34)	50.3 (3.14)	41.7 (1.33)	37.9 (1.42)	39.2 (2.50)
Estimated TDN^2)^	82.9	83.4	82.8	83.5	81.3
Microbial population^3)^					
Total bacteria	4.88	5.22	4.21	4.55	5.01
Yeast	3.42	3.78	<2.8	3.22	3.11
Mold	3.37	3.02	<2.8	<2.8	3.51
Lactic acid bacteria	<2.8	<2.8	3.11	<2.8	<2.8

DM, dry matter; NDF, neutral detergent fiber; ADF, acid detergent fiber; NFC, non-fibrous carbohydrates; WSC, water-soluble carbohydrates; TDN, total digestible nutrients.

1) Fruit and vegetable discards were collected every 20 d from Emart Fresh Center, and those ingredients constituting over 90% of total discards were selected. Sodium metabisulfite was added at 6 g/kg of fruit and vegetable discards (wet basis). The treated discards were stored outside for 1 wk under aerobic conditions before being mixed with other TMR ingredients.

2) TDN was predicted through the equation of Conrad et al [30].

3) Expressed as logarithm number of colony-forming units/g fresh mass. Limit of detection = 2.8 log_10_ cfu/g fresh mass.

**Table 2:** Analyzed chemical composition (% of DM, unless otherwise stated) of individual ingredients used in dietary treatments (Exp. 1)

Feed ingredients	DM (%)	NDF	ADF	EE	CP	Ash	NFC
Rice straw	58.6	72.8	42.5	0.99	3.78	11.9	10.5
Alfalfa hay	89.5	30.1	23.8	2.01	16.4	6.35	45.1
Tall fescue hay	90.8	73.1	44.8	1.02	5.23	4.99	15.7
Wet brewers grain	25.4	65.4	37.8	4.77	22.7	4.15	2.98
Corn grain, cracked	87.1	12.9	8.22	3.33	7.81	1.42	74.5
Soybean meal	88.9	31.9	16.7	1.79	43.8	6.67	15.8

DM, dry matter; NDF, neutral detergent fiber; ADF, acid detergent fiber; EE, ether extract; CP, crude protein; NFC, non-fibrous carbohydrates.

**Table 3:** Ingredient and chemical composition of diets fed to Hanwoo heifers (Exp. 1; values in parentheses indicate standard deviation)

Items	Level of SMB-treated FVD, % of as-fed^1)^		

	0	10	20
Ingredients, as-fed basis			
Wet brewers grain	39.5	29.5	19.6
Rice straw	20.5	20.5	20.5
Tall fescue hay	14.0	14.0	14.0
Alfalfa hay	7.0	7.0	7.0
Corn grain, cracked	14.0	14.0	13.9
Soybean meal	4.31	4.30	4.30
SMB-treated FVD	0.0	10.0	20.0
Salt	0.25	0.25	0.25
Limestone	0.30	0.30	0.30
Vitamin/mineral premix^2)^	0.10	0.10	0.10
Chemical composition^3)^			
DM (%)	58.5 (1.34)	57.1 (2.63)	56.6 (2.96)
NDF (% of DM)	45.0 (1.18)	43.1 (2.07)	42.6 (1.31)
ADF (% of DM)	27.1 (2.14)	25.7 (1.95)	25.1 (2.28)
Lignin (% of DM)	5.97 (0.42)	5.92 (0.59)	5.88 (1.03)
Ether extract (% of DM)	2.81 (0.21)	2.63 (0.32)	2.49 (0.36)
Crude protein (% of DM)	13.3 (0.38)	12.8 (0.41)	12.5 (0.52)
Ash (% of DM)	8.40 (0.83)	8.58 (0.36)	8.49 (0.75)
NFC (% of DM)	29.9 (0.60)	32.4 (0.74)	33.9 (0.69)
WSC (% of DM)	5.19 (0.71)	6.34 (0.89)	7.52 (0.94)
WSC:crude protein ratio	0.39	0.50	0.60
Calcium^4)^ (% of DM)	0.40	0.44	0.45
Phosphorus^4)^ (% of DM)	0.33	0.31	0.30
TDN^4)^ (% of DM)	60.1	60.8	59.9

SMB, sodium metabisulfite; FVD, fruit and vegetable discards; DM, dry matter; NDF, neutral detergent fiber; ADF, acid detergent fiber; NFC, non-fibrous carbohydrates; WSC, water-soluble carbohydrates; TDN, total digestible nutrients.

1) SMB-treated FVD = sodium metabisulfite was added 6 g/kg of fruit and vegetable discards (wet basis). The treated discards were stored outside for 1 wk under aerobic conditions. Diets containing the different levels of SMB-treated FVD were prepared in the form of TMR every 3 wk and stored anaerobically in sealed bags (200 kg).

2) Contained per kg of premix: vitamin A (1,000,000 IU), vitamin D_3_ (200,000 IU), vitamin E (250 mg), vitamin B_1_ (410 mg), vitamin B_2_ (400 mg), vitamin B_12_ (5 mg), niacin (500 mg), folic acid (450 mg); methionine (5 g), β-carotene (1.1 g), Zn (2.1 g), Cu (1.7 g), Mn (1.55 g), Fe (1.5 g), and Se (2.15 mg).

3) Calculated based on the analyzed chemical composition of individual ingredients.

4) Based on tabular values reported in the Korean Feeding Standard Establishment Council (2007). The equation of Conrad et al [30] was used for TDN estimation of FVD.

**Table 4:** Treatment effects on feed intake and growth rate of Hanwoo heifers (Exp. 1; n = 12/treatment)

Items	Level of SMB-treated FVD, % of as-fed^1)^			SEM	p-value		
	
	0	10	20		Treatment	Linear	Quadratic
Daily DM intake (kg)	6.78	6.61	6.34	0.61	0.31	0.19	0.54
Initial body weight (kg)	305	304	306	4.52	0.97	0.94	0.89
Final body weight (kg)	363	358	361	5.75	0.89	0.75	0.73
Total weight gain (kg)	57.3	54.8	55.5	5.31	0.93	0.79	0.81
Average daily gain (kg)	0.64	0.61	0.62	0.07	0.74	0.65	0.34
Feed conversion ratio^2)^	10.6	10.8	10.5	0.69	0.51	0.43	0.20
Final body condition score	3.54	3.41	3.50	0.07	0.42	0.68	0.22

SMB, sodium metabisulfite; FVD, fruit and vegetable discards; SEM, standard error of mean; DM, dry matter.

1) SMB-treated FVD = sodium metabisulfite was added at 6 g/kg of fruit and vegetable discards (wet basis). The treated discards were stored outside for 1 wk under aerobic conditions until being added to TMR.

2) Feed conversion ratio = daily DM consumed (kg)/average daily gain (kg).

**Table 5:** Treatment effects on blood chemistry and hematological parameters of Hanwoo heifers (Exp. 1; n = 12/treatment)

Items	Level of SMB-treated FVD, % of as-fed^1)^			SEM	p-value		
	
	0	10	20		Treatment	Linear	Quadratic
Blood chemistry							
Total protein (g/dL)	6.93	7.16	6.89	0.15	0.14	0.79	0.06
Globulin (g/dL)	2.83	3.05	2.94	0.16	0.49	0.63	0.28
Albumin (g/dL)	4.10	4.11	3.94	0.08	0.17	0.18	0.18
Creatinine (mg/dL)	1.31	1.26	1.23	0.10	0.71	0.42	0.91
Urea-N (mg/dL)	14.3	13.9	11.1	0.66	<0.01	<0.01	0.18
Glucose (mg/dL)	59.5	55.9	62.4	1.95	0.08	0.31	0.06
Cholesterol (mg/dL)	147	147	138	9.90	0.77	0.55	0.69
Free fatty acids (μEq/L)	163	148	157	12.7	0.72	0.75	0.46
High-density lipoprotein (mg/dL)	125	105	115	6.90	0.16	0.29	0.11
Low-density lipoprotein (mg/dL)	55.0	56.1	49.6	5.66	0.69	0.51	0.59
Ceruloplasmin (mg/dL)	5.19	5.13	4.80	0.30	0.64	0.38	0.73
Total ketones (μmol/L)	607	585	461	40.6	0.04	0.02	0.32
Acetoacetate (μmol/L)	45.7	44.1	33.8	2.30	<0.01	<0.01	0.14
β-hydroxybutyric acid (μmol/L)	562	541	427	39.2	0.05	0.02	0.34
Blood mineral							
Inorganic phosphorus (mg/dL)	8.44	8.49	8.30	0.19	0.79	0.63	0.63
Sodium (mmol/L)	145	144	142	0.69	<0.01	<0.01	0.56
Calcium (mg/dL)	10.4	10.5	10.3	0.14	0.59	0.95	0.31
Chlorine (mmol/L)	102	102	100	0.86	0.35	0.16	0.73
Copper (μg/dL)	71.3	73.9	67.4	4.06	0.53	0.51	0.37
Iron (μg/dL)	194	179	210	10.9	0.38	0.79	0.18
Organ function							
Alanine aminotransferase (U/L)	26.9	28.3	27.4	1.46	0.80	0.81	0.54
Alkaline phosphatase (U/L)	144	148	151	11.1	0.44	0.21	0.53
Aspartate aminotransferase (U/L)	65.3	78.0	68.8	7.89	0.51	0.76	0.27
γ-glutamyltransferase (U/L)	14.9	13.3	13.1	2.24	0.68	0.43	0.69
Creatinine phosphokinase (U/L)	223	215	246	42.3	0.76	0.60	0.60
Hematology							
White blood cell (10^3^/μL)	8.64	8.57	8.78	0.29	0.96	0.86	0.83
Red blood cell (10^6^/μL)	8.31	8.50	8.59	0.14	0.71	0.42	0.89
Hematocrit (%)	38.4	38.2	37.8	0.54	0.92	0.70	0.89
Hemoglobin (g/dL)	12.9	12.6	12.7	0.19	0.84	0.68	0.67
Mean corpuscular volume (fL)	46.5	45.2	44.1	0.61	0.31	0.13	0.92
Mean cell hemoglobin (g/dL)	15.6	14.9	14.8	0.21	0.27	0.15	0.49
MCHC (g/dL)	33.6	32.9	33.5	0.18	0.26	0.93	0.11
Platelets (10^3^/μL)	482	483	494	18.5	0.96	0.79	0.90

SMB, sodium metabisulfite; FVD, fruit and vegetable discards; SEM, standard error of mean; MCHC, mean corpuscular hemoglobin concentration.

1) SMB-treated FVD = sodium metabisulfite was added at 6 g/kg of fruit and vegetable discards (wet basis). The treated discards were stored outdoor under aerobic conditions for 1 wk until being added to TMR.

**Table 6:** Treatment effects on behavior of Hanwoo heifers during daylight (Exp. 1; 07:00 to 18:00 h; n = 12/treatment)

Items	Level of SMB-treated FVD, % of as-fed^1)^			SEM	p-value		
	
	0	10	20		Treatment	Linear	Quadratic
Standing^2)^ (min)	578	582	572	21.5	0.42	0.53	0.33
Rumination^3)^ (min)	101	99.2	88.3	11.2	0.17	0.12	0.21
Lying (min)	82.1	78.2	88.1	5.12	0.53	0.56	0.18
Eating (min)	155	163	147	16.7	0.09	0.14	0.06
Chewing^4)^ (min)	256	262	235	20.8	0.13	0.28	0.11
Drinking frequency	10.3	9.15	11.3	3.11	0.45	0.52	0.13
Urinating frequency	4.56	4.22	5.11	0.96	0.31	0.39	0.21
Defecating frequency	6.45	5.17	6.11	1.28	0.52	0.46	0.19

SMB, sodium metabisulfite; FVD, fruit and vegetable discards; SEM, standard error of mean.

1) SMB-treated FVD = sodium metabisulfite was added at 6 g/kg of fruit and vegetable discards (wet basis). The treated discards were stored outside for 1 wk under aerobic conditions until being added to TMR.

2) Standing time includes eating time.

3) Rumination was recorded during both standing and lying states.

4) Chewing time = sum of eating time and rumination time.

**Table 7:** Ingredient and chemical composition of sodium metabisulfite-treated fruit and vegetable discards used in dietary treatments (Exp. 2; values in parentheses indicate standard deviation)

Items	Collection days^1)^			

	d 0	d 20	d 40	d 60
Individual ingredients (%)				
Grape	23.7	8.46	6.81	9.09
Lemon	35.6	2.51	9.70	9.78
Onion	18.0	15.4	4.11	8.31
Pumpkin	9.88	3.21	0	4.21
Paprika	5.93	10.5	6.71	14.2
Melon	3.95	0	19.4	9.33
Sweet potato	2.94	0	2.54	3.20
Orange	0	36.8	15.2	4.07
Apple	0	14.4	20.8	11.1
Tomato	0	6.41	7.60	5.21
Green onion stalk	0	2.31	0	0
Persimmon	0	0	2.32	0
Watermelon	0	0	0	10.4
Plum	0	0	4.81	11.1
Nutrient composition				
DM (%)	14.2 (0.21)	15.0 (0.19)	14.1 (0.31)	15.6 (0.18)
NDF (% of DM)	15.7 (0.52)	17.7 (0.34)	15.4 (0.31)	16.6 (0.44)
ADF (% of DM)	10.8 (0.13)	12.4 (0.11)	11.2 (0.09)	11.4 (0.19)
Lignin (% of DM)	3.23 (0.12)	2.09 (0.11)	2.11 (0.07)	2.75 (0.10)
Ether extract (% of DM)	1.15 (0.09)	2.25 (0.08)	2.00 (0.13)	1.84 (0.09)
Crude protein (% of DM)	7.62 (0.62)	7.36 (0.30)	6.51 (0.36)	7.19 (0.49)
Ash (% of DM)	4.03 (0.31)	4.10 (0.18)	4.44 (0.31)	4.64 (0.12)
NFC (% of DM)	71.5 (0.56)	68.6 (0.81)	71.7 (0.45)	69.7 (0.58)
NH_3_-N (% of total N)	11.2 (0.45)	7.11 (0.78)	10.3 (0.31)	8.67 (0.56)
WSC (% of DM)	49.9 (3.11)	56.2 (2.09)	53.4 (3.11)	48.8 (2.78)
Estimated TDN^2)^	81.4	83.8	83.7	82.2

SMB, sodium metabisulfite; FVD, fruit and vegetable discards; DM, dry matter; NDF, neutral detergent fiber; ADF, acid detergent fiber; NFC, non-fibrous carbohydrates; WSC, water-soluble carbohydrates; TDN, total digestible nutrients.

1) Fruit and vegetable discards were collected every 20 d from Emart Fresh Center, and those ingredients constituting over 90% of total discards were selected. Sodium metabisulfite was added at 6 g/kg of fruit and vegetable discards (wet basis). The treated discards were stored outside for 1 wk under aerobic conditions before being mixed with other TMR ingredients.

2) The TDN content of FVD was estimated using the equation of Conrad et al [30].

**Table 8:** Ingredient and chemical composition diets fed to Hanwoo cows (Exp. 2; values in parentheses indicate standard deviation)

Items	Level of SMB-treated FVD, % of as-fed^1)^		

	0	11	22
Ingredients, as-fed basis			
Wet brewers grain	33.0	22.0	11.0
Barley straw	13.0	13.0	13.0
Ryegrass hay	13.0	13.0	13.0
Alfalfa hay	10.0	10.0	10.0
Corn grain, cracked	18.0	18.0	18.0
Soybean meal	6.00	6.00	6.00
Beet pulp pellet	5.60	5.60	5.60
SMB-treated FVD	0.0	11.0	22.0
Salt	0.20	0.20	0.20
Limestone	0.60	0.60	0.60
Vitamin/mineral premix^2)^	0.30	0.30	0.30
Yeast culture	0.30	0.30	0.30
Chemical composition^3)^			
DM (%)	63.6 (2.89)	62.8 (3.01)	61.7 (1.98)
NDF (% of DM)	37.3 (1.90)	36.9 (2.33)	36.4 (2.17)
ADF (% of DM)	24.1 (1.56)	24.0 (2.11)	24.0 (1.67)
Ether extract (% of DM)	2.68 (0.18)	2.54 (0.33)	2.40 (0.24)
Crude protein (% of DM)	13.5 (0.51)	12.9 (0.41)	12.4 (0.44)
Ash (% of DM)	6.25 (0.23)	6.34 (0.42)	6.44 (0.33)
Forage DM (% of DM)	46.8 (1.79)	47.5 (2.13)	48.3 (2.76)
NFC (% of DM)	40.3 (0.89)	41.3 (1.01)	42.4 (0.69)
WSC (% of DM)	4.67 (0.45)	6.22 (0.77)	7.82 (0.63)
WSC:crude protein ratio	0.35	0.48	0.63
Calcium^4)^ (% of DM)	0.68	0.69	0.69
Phosphorus^4)^ (% of DM)	0.36	0.35	0.34
TDN^4)^ (% of DM)	64.7	64.8	64.6

SMB, sodium metabisulfite; FVD, fruit and vegetable discards; DM, dry matter; NDF, neutral detergent fiber; ADF, acid detergent fiber; NFC, non-fibrous carbohydrates; WSC, water-soluble carbohydrates; TDN, total digestible nutrients.

1) SMB-treated FVD = sodium metabisulfite was added 6 g/kg of fruit and vegetable discards (wet basis). The treated discards were stored outside for 1 wk under aerobic conditions. Diets containing the different levels of SMB-treated FVD were prepared in the form of TMR every 3 wk and stored anaerobically in sealed bags (200 kg).

2) Contained per kg of premix: vitamin A (2,500,000 IU), vitamin D_3_ (500,000 IU), vitamin E (1,000 IU), Fe (5.6 g), Mn (4 g), Mg (3 g), Zn (1.5 g), Cu (375 mg), I (140 mg), and Co (100 mg).

3) Calculated based on the analyzed chemical composition of individual ingredients.

4) Based on tabular values reported in the Korean Feeding Standard Establishment Council (2007). The equation of Conrad et al [30] was used for TDN estimation of FVD.

**Table 9:** Analyzed chemical composition (% of DM, unless otherwise stated) of individual ingredients used in dietary treatments (Exp. 2)

Feed ingredients	DM (%)	NDF	ADF	EE	CP	Ash	NFC
Alfalfa hay	87.3	32.5	24.3	2.41	18.2	5.67	41.2
Wet brewers grain	23.7	67.4	35.8	4.27	21.7	4.52	2.11
Corn grain, cracked	89.1	12.1	9.43	3.53	7.61	1.67	75.1
Soybean meal	87.4	33.2	14.9	1.65	44.2	6.45	14.5
Barley straw	62.3	79.1	65.1	0.88	3.39	11.2	5.43
Ryegrass hay	88.5	73.6	44.9	0.82	6.34	4.08	15.2
Beet pulp pellet	90.5	48.1	25.1	1.11	10.3	3.82	36.7

DM, dry matter; NDF, neutral detergent fiber; ADF, acid detergent fiber; EE, ether extract; CP, crude protein; NFC, non-fibrous carbohydrates.

**Table 10:** Treatment effects on daily DM intake and body condition score of Hanwoo cows before and after experiment (Exp. 2; n = 8/treatment)

Items	Level of SMB-treated FVD, % of as-fed^1)^			SEM	p-value		
	
	0	11	22		Treatment	Linear	Quadratic
Daily DM intake (kg)	8.39	8.27	8.14	0.72	0.62	0.28	0.69
Body condition score							
Initial	2.56	2.69	2.66	0.08	0.52	0.41	0.43
Final	2.63	2.81	2.79	0.11	0.39	0.19	0.58
Difference	0.07	0.12	0.13	0.02	0.31	0.21	0.39

DM, dry matter; SMB, sodium metabisulfite; FVD, fruit and vegetable discards; SEM, standard error of mean; TMR, total mixed ration.

1) SMB-treated FVD = sodium metabisulfite was added at 6 g/kg of fruit and vegetable discards (wet basis). The treated discards were stored outside for 1 wk under aerobic conditions until being added to TMR.

**Table 11:** Treatment effects on blood chemistry and hematological parameters of Hanwoo cows (Exp. 2; n = 8/treatment)

Items	Level of SMB-treated FVD, % of as-fed^1)^			SEM	p-value		
	
	0	11	22		Treatment	Linear	Quadratic
Blood chemistry							
Total protein (g/dL)	7.03	7.20	7.23	0.11	0.78	0.51	0.80
Globulin (g/dL)	3.48	3.52	3.67	0.10	0.78	0.51	0.81
Albumin (g/dL)	3.55	3.68	3.57	0.05	0.51	0.89	0.26
Creatinine (mg/dL)	1.32	1.33	1.35	0.05	0.97	0.81	0.95
Urea-N (mg/dL)	14.9	12.4	12.3	0.48	0.05	0.03	0.19
Glucose (mg/dL)	68.2	67.0	67.3	1.29	0.94	0.81	0.80
Cholesterol (mg/dL)	139	131	135	5.15	0.81	0.73	0.59
Free fatty acids (μEq/L)	73.0	74.8	85.5	4.53	0.41	0.33	0.83
High-density lipoprotein (mg/dL)	110	113	116	3.76	0.86	0.59	0.95
Low-density lipoprotein (mg/dL)	49.0	44.1	43.8	2.53	0.69	0.45	0.70
Ceruloplasmin (mg/dL)	5.82	5.72	6.10	1.07	0.84	0.67	0.68
Total ketones (μmol/L)	676	753	604	128	0.14	0.33	0.09
Acetoacetate (μmol/L)	69.6	76.5	63.4	15.1	0.36	0.50	0.22
β-hydroxybutyric acid (μmol/L)	607	641	541	23.5	0.27	0.28	0.23
Blood mineral							
Inorganic phosphorus (mg/dL)	6.37	6.25	5.73	0.19	0.38	0.19	0.63
Sodium (mmol/L)	141	142	141	0.26	0.47	0.46	0.32
Calcium (mg/dL)	10.0	10.2	10.2	0.09	0.60	0.34	0.76
Chlorine (mmol/L)	99.5	99.7	97.0	0.04	<0.01	<0.01	0.04
Copper (μg/dL)	70.1	77.0	82.8	2.29	0.17	0.06	0.97
Iron (μg/dL)	142	156	156	4.32	0.34	0.22	0.43
Organ function							
Alanine aminotransferase (U/L)	32.0	26.7	31.3	1.37	0.29	0.85	0.13
Alkaline phosphatase (U/L)	66.2	57.3	67.2	3.98	0.85	0.93	0.59
Aspartate aminotransferase (U/L)	65.8	58.5	70.5	3.06	0.31	0.56	0.17
γ-glutamyltransferase (U/L)	19.7	18.0	18.1	1.03	0.20	0.20	0.21
Creatinine phosphokinase (U/L)	166	126	222	18.8	0.16	0.23	0.11
Hematology							
White blood cell (10^3^/μL)	9.05	7.90	8.55	1.34	0.28	0.31	0.22
Red blood cell (10^6^/μL)	7.12	6.65	7.11	0.92	0.63	0.98	0.35
Hematocrit (%)	32.9	33.4	34.5	3.13	0.71	0.43	0.86
Hemoglobin (g/dL)	12.3	12.5	12.5	1.19	0.94	0.79	0.86
Mean corpuscular volume (fL)	46.6	50.3	48.8	0.78	0.17	0.26	0.13
Mean cell hemoglobin (g/dL)	17.5	18.8	17.7	0.37	0.31	0.82	0.14
MCHC (g/dL)	37.5	37.4	36.3	0.30	0.21	0.11	0.43
Platelets (10^3^/μL)	451	440	500	23.7	0.39	0.43	0.27

SMB, sodium metabisulfite; FVD, fruit and vegetable discards; SEM, standard error of mean; MCHC, mean corpuscular hemoglobin concentration; TMR, total mixed ration.

1) SMB-treated FVD = sodium metabisulfite was added at 6 g/kg of fruit and vegetable discards (wet basis). The treated discards were stored outside for 1 wk under aerobic conditions until being added to TMR.

**Table 12:** Treatment effects on behavior of Hanwoo cows during daylight (07:00 to 18:00 h; Exp. 2; n = 8/treatment)

Items	Level of SMB-treated FVD, % of as-fed^1)^			SEM	p-value		
	
	0	11	22		Treatment	Linear	Quadratic
Standing time^2)^ (min)	540	519	529	22.4	0.65	0.58	0.20
Rumination time^3)^ (min)	111	116	101	13.2	0.24	0.19	0.11
Lying time (min)	120	141	131	11.2	0.27	0.24	0.13
Eating time (min)	203	196	206	10.2	0.16	0.21	0.09
Chewing time^4)^ (min)	314	312	307	18.7	0.44	0.56	0.38
Drinking frequency	8.31	8.89	9.30	2.01	0.52	0.61	0.27
Urinating frequency	5.45	4.21	4.33	1.12	0.38	0.33	0.21
Defecating frequency	5.45	6.17	5.66	1.32	0.52	0.49	0.18

SEM, standard error of mean.

1) SMB-treated FVD = sodium metabisulfite was added at the rate of 6 g/kg of fruit and vegetable discards (wet basis). The treated discards were stored outside for 1 wk under aerobic conditions until being added to TMR.

2) Standing time includes eating time.

3) Rumination was recorded during both standing and lying states.

4) Chewing time = sum of eating and rumination time.

## References

[1] Sagar NA, Pareek S, Sharma S, Yahia EM, Lobo MG (2018). Fruit and vegetable waste: bioactive compounds, their extraction, and possible utilization. Compr Rev Food Sci Food Saf.

[2] Opara UL, Pathare PB (2014). Bruise damage measurement and analysis of fresh horticultural produce–a review. Postharvest Biol Technol.

[3] Ahmadi F, Lee YH, Lee WH, Oh YK, Park KK, Kwak WS (2018). Preservation of fruit and vegetable discards with sodium metabisulfite. J Environ Manage.

[4] Ahmadi F, Lee WH, Oh YK, Park K, Kwak WS (2020). Fruit and vegetable discards preserved with sodium metabisulfite as a high-moisture ingredient in total mixed ration for ruminants: effect on in vitro ruminal fermentation and in vivo metabolism. Asian-Australas J Anim Sci.

[5] Romero-Huelva M, Ramos-Morales E, Molina-Alcaide E (2012). Nutrient utilization, ruminal fermentation, microbial abundances, and milk yield and composition in dairy goats fed diets including tomato and cucumber waste fruits. J Dairy Sci.

[6] Ahmadi F, Lee WH, Oh YK, Park K, Kwak WS (2020). Microbial, nutritional, and antioxidant stability of fruit and vegetables discards treated with sodium metabisulfite during aerobic and anaerobic storage. Waste Biomass Valorization.

[7] Ahmadi F, Lee WH, Oh YK, Park KK, Kwak WS (2019). Long-term anaerobic conservation of fruit and vegetable discards without or with moisture adjustment after aerobic preservation with sodium metabisulfite. Waste Manag.

[8] Wang F, Nishino N (2008). Ensiling of soybean curd residue and wet brewers grains with or without other feeds as a total mixed ration. J Dairy Sci.

[9] Murdock FR, Hodgson AS, Riley RE (1981). Nutritive value of wet brewers grains for lactating dairy cows. J Dairy Sci.

[10] Dubois M, Gilles KA, Hamilton JK, Rebers P, Smith F (1956). Colorimetric method for determination of sugars and related substances. Anal Chem.

[11] Barker SB, Summerson WH (1941). The colorimetric determination of lactic acid in biological material. J Biol Chem.

[12] AOAC International (2012). Official methods of analysis.

[13] Westendorf ML, Wohlt JE (2002). Brewing by-products: their use as animal feeds. Vet Clin North Am Food Anim Pract.

[14] Song KH, Woo JS, Kim JR (2020). Nutritional value and in situ degradability of fruit-vegetable byproducts and their feeding effects on performance of growing Hanwoo steers. Asian-Australas J Anim Sci.

[15] Cordukes WE, Shearer DA (1961). Nutrient preservation and acceptability of forages ensiled with sodium metabisulphite at varying levels of compaction and dry matter. Can J Plant Sci.

[16] Smith WC, Campbell IL (1960). Sodium metabisulphite as an additive in silage making. NZ J Agric Res.

[17] Lee MRF, Jones EL, Moorby JM, Humphreys MO, Theodorou MK, Scollan ND (2001). Production responses from lambs grazed on Lolium perenne selected for an elevated water-soluble carbohydrate concentration. Anim Res.

[18] Sutoh M, Obara Y, Miyamoto S (1996). The effect of sucrose supplementation on kinetics of nitrogen, ruminal propionate and plasma glucose in sheep. J Agric Sci (Camb).

[19] Choe C, Jun YH, Do YJ (2018). Hematological analysis of the Korean native cattle (Hanwoo) according to the period and method of grazing. Korean J Vet Serv.

[20] Hoover WH, Stokes SR (1991). Balancing carbohydrates and proteins for optimum rumen microbial yield. J Dairy Sci.

[21] Osborne VR, Leslie KE, McBride BW (2002). Effect of supplementing glucose in drinking water on the energy and nitrogen status of the transition dairy cow. Can J Anim Sci.

[22] Broderick GA, Luchini ND, Reynal SM, Varga GA, Ishler VA (2008). Effect on production of replacing dietary starch with sucrose in lactating dairy cows. J Dairy Sci.

[23] Edwards GR, Parsons AJ, Rasmussen S, Bryant RH High sugar ryegrasses for livestock systems in New Zealand.

[24] Butler ST, Pelton SH, Butler WR (2006). Energy balance, metabolic status, and the first postpartum ovarian follicle wave in cows administered propylene glycol. J Dairy Sci.

[25] Grummer RR, Winkler JC, Bertics SJ, Studer VA (1994). Effect of propylene glycol dosage during feed restriction on metabolites in blood of prepartum Holstein heifers. J Dairy Sci.

[26] Gengelbach GP, Ward JD, Spears JW (1994). Effect of dietary copper, iron, and molybdenum on growth and copper status of beef cows and calves. J Anim Sci.

[27] Meiske JC, Prouty RM, Schuman LM, Scaletti JV (1965). Effect of sodium bisulfite additions to corn silages. J Anim Sci.

[28] Eversole DE, Browne MF, BHJ, Dietz RE (c2009). Body condition scoring beef cows.

[29] Roland L, Drillich M, Iwersen M (2014). Hematology as a diagnostic tool in bovine medicine. J Vet Diagn Invest.

[30] Conrad HR, Weiss WP, Odwongo WO, Shockey WL (1984). Estimating net energy lactation from components of cell solubles and cell walls. J Dairy Sci.

